# Estrogen Signaling in Endometrial Cancer: a Key Oncogenic Pathway with Several Open Questions

**DOI:** 10.1007/s12672-019-0358-9

**Published:** 2019-02-02

**Authors:** Adriana C. Rodriguez, Zannel Blanchard, Kathryn A. Maurer, Jason Gertz

**Affiliations:** 1 Huntsman Cancer Institute, University of Utah, Salt Lake City, UT USA; 2 Department of Oncological Sciences, University of Utah School of Medicine, Salt Lake City, UT USA; 3 Department of Obstetrics and Gynecology, University of Utah School of Medicine, Salt Lake City, UT USA

**Keywords:** Endometrial cancer, Estrogen receptor alpha, Estrogen signaling, Progesterone signaling, Gene regulation

## Abstract

Endometrial cancer is the most common gynecological cancer in the developed world, and it is one of the few cancer types that is becoming more prevalent and leading to more deaths in the USA each year. The majority of endometrial tumors are considered to be hormonally driven, where estrogen signaling through estrogen receptor α (ER) acts as an oncogenic signal. The major risk factors and some treatment options for endometrial cancer patients emphasize a key role for estrogen signaling in the disease. Despite the strong connections between estrogen signaling and endometrial cancer, important molecular aspects of ER function remain poorly understood; however, progress is being made in our understanding of estrogen signaling in endometrial cancer. Here, we discuss the evidence for the importance of estrogen signaling in endometrial cancer, details of the endometrial cancer-specific actions of ER, and open questions surrounding estrogen signaling in endometrial cancer.

## Introduction

Uterine cancer is the fourth most common cancer in women, and the most common gynecological cancer, accounting for more than 60,000 cases and 10,000 deaths in the USA each year [1]. Endometrial cancer is the most common type of uterine cancer and is subdivided into two types by histopathology [2]. Type I endometrial tumors, also known as low grade endometrioid, make up the majority of endometrial cancer cases (~ 85%), are low grade with a glandular structure, usually express high levels of estrogen receptor α (ER), and are thought to be hormonally driven [3]. Type II tumors include high-grade endometrioid tumors, serous tumors, clear cell tumors, carcinosarcomas, and tumors with mixed histology. These tumors are less likely to express ER [4, 5], have a worse prognosis, and share molecular features with triple-negative breast cancer and serous ovarian cancer, including a high prevalence of p53 mutations and high copy number variation [6]. Despite the worse prognosis of type II endometrial cancers, the hormonally driven type I endometrial cancers are responsible for more deaths because of their increased incidence [7].

Recent in-depth molecular analysis of large collections of endometrial tumors has provided additional resolution to the subtypes of the disease [8]. The Cancer Genome Atlas (TCGA) project integrated mutational analysis, copy number variation, and mRNA expression to identify four molecular subtypes: ultramutated—defined by *POLE* mutations, microsatellite instable, copy-number low, and copy-number high. Tumors from each of the four subtypes express high levels of ER, with the exception of copy-number high, which are type II endometrial tumors [8]. The histological and molecular subtypes of endometrial cancer both point to the potential for estrogen signaling through ER to be active in the majority of endometrial tumors. In this review, we discuss both the evidence that estrogen signaling plays a central role in endometrial cancer as well as the uncovered and yet to be discovered molecular details surrounding ER in the disease.

## Estrogen-Associated Risk Factors for Endometrial Cancer

Estrogens play a mitogenic role in the normal endometrium, driving tissue growth as part of pregnancy anticipation during the menstrual cycle. During the late follicular phase of the menstrual cycle, estrogens (particularly estrone and 17β-estradiol, or E1 and E2, respectively) are produced by the developing follicle leading to growth of the endometrium [9]. Estrogen production peaks at ovulation, the end of the follicular phase, but is produced at lower levels by the corpus luteum during the mid- and late-luteal phase before dropping prior to menstruation [10]. The second wave of estrogens does not lead to endometrial cell proliferation, and this is due to the presence of progestogens, particularly progesterone. Progesterone levels are low during the follicular phase of the menstrual cycle but rise due to corpus luteum production in the mid- and late-luteal phase [11]. Progesterone inhibits estrogen-induced endometrial growth during the luteal phase while also transitioning the endometrium to a receptive state that is ready for blastocyst implantation. This balance between pro-growth estrogens and anti-growth progestogens is often dominated by estrogens during cancer formation. In animal models, high levels of estrogens unopposed by progesterone lead to endometrial hyperplasia or cancer [12–15], suggesting that the lack of estrogen/progesterone balance can contribute to the early stages of endometrial cancer formation.

**Fig. 1:**
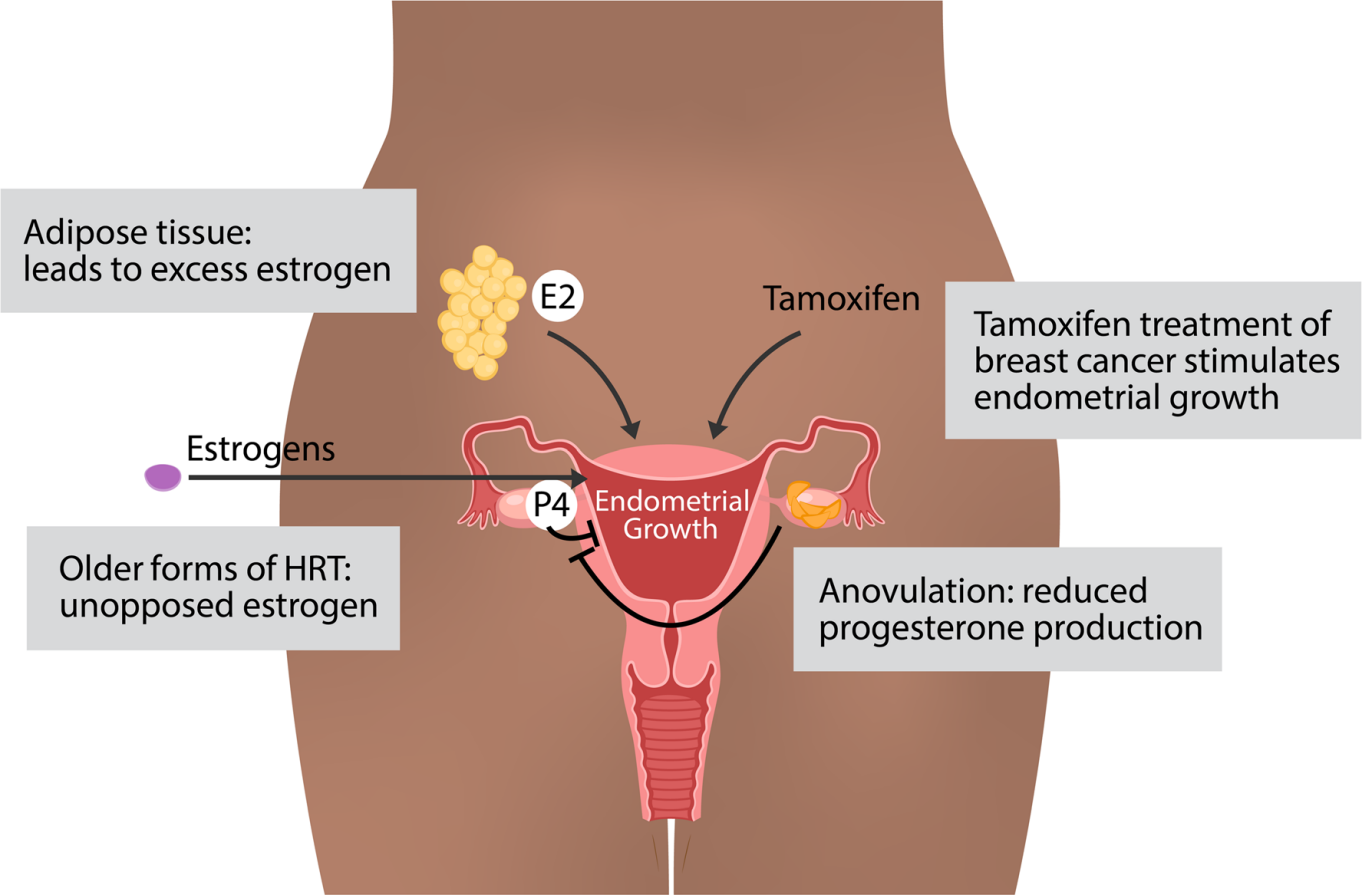
Risk factors for endometrial cancer. In general, estrogens, including 17β-estradiol (E2), drive endometrial growth and progestogens, including progesterone (P4), block endometrial growth and promote differentiation. Excess estrogens can be caused by obesity, where adipose tissue can synthesize estrogens, estrogen only hormone replacement therapy (HRT), and breast cancer treatment with tamoxifen, which acts as a partial ER agonist in endometrial cells. Loss of progesterone, which can occur due to anovulation, can also contribute to endometrial growth

Consistent with the fundamental role of estrogens and progestogens in growth of the endometrium, many of the endometrial cancer risk factors involve excess estrogens or estrogen signaling unopposed by progesterone signaling (Fig. 1). One of the most important and prevalent risk factors for endometrial cancer is obesity. Obese women have a 3-fold increased risk of developing endometrial cancer [16]. In a recent umbrella review of risk factors and endometrial cancer incidence, body mass index was strongly associated with increased cancer risk in premenopausal women (relative risk per 5 kg/m^2^ = 1.49) and postmenopausal women (relative risk per 5 kg/m^2^ = 1.60) [17]. The link between estrogens and obesity stems from adipose tissue’s ability to synthesize estrogen [18]. In adipose tissue, both adipocytes and stromal cells express aromatase [19], the enzyme responsible for converting androgens to estrogens [20]. The excess estrogens produced by the adipose tissue provides a growth signal for the endometrium that is unopposed by progesterone. Obesity also leads to higher rates of anovulation [21] with a relative risk above 2 for body mass index greater than 29 [22]; however, most obese women have normal ovulatory menstrual cycles [23]. In anovulatory women, the lack of ovulation and corpus luteum production keeps progesterone levels low. Diminished progesterone is unable to execute important growth suppression of the estrogen stimulated endometrium [24], leading to unopposed growth of endometrial cells. Patients with polycystic ovarian syndrome (PCOS) have an increased risk of endometrial cancer [25], and part of this risk likely relates to anovulation which again leads to estrogen signaling that is unopposed by progesterone signaling. Obesity is a risk factor for PCOS, although many nonobese women develop PCOS and many obese women do not exhibit PCOS. PCOS is also more complex than simply a reduction in progesterone; progesterone levels are not always reduced and the levels of other hormones can be affected [26]. In addition to obesity and PCOS, other risk factors include estrogen therapy without progestins [27], tamoxifen for the treatment of breast cancer (discussed below), parity, oral contraceptive use, cigarette smoking, age at menarche, and diabetes [7]. With the exception of genetic predisposition syndromes, such as Lynch syndrome [28] and Cowden syndrome [29], risk factors implicate estrogen signaling as a key driving force in endometrial cancer formation.

## Hormone Therapy for the Treatment of Endometrial Cancer

The most common symptom of endometrial cancer is abnormal vaginal bleeding [30], which leads to most endometrial tumors being diagnosed at an early stage. Because of the early detection, surgical removal of the uterus and ovaries for type I endometrial tumors is very effective with only a minority of patients requiring adjuvant treatment, most commonly vaginal brachytherapy [31]. In the subset of patients with type I endometrial cancer who are either unable to undergo surgery or want to maintain fertility, progestins (synthetic progestogens) are given as the main course of treatment. Progestins work by binding to and activating progesterone receptor (PR). As discussed above, progestogens block estrogen-induced uterine growth and PR is thought to block the pro-growth actions of ER in a cell autonomous fashion in breast cancer cells by binding to similar sites and altering gene regulation [32]; however, it is unclear if a similar mechanism underlies the opposing effects of ER and PR in endometrial cancer cells. Progestin therapies have a strong initial response (~ 75%), and approximately half of young patients desiring uterine-sparing treatment will be cancer free in the long term when treated with progestins alone [33, 34].

Despite the promising results of progestins for patients receiving uterine-sparing treatment, adjuvant hormone therapy has not been shown to confer benefit to endometrial cancer patients after surgery [35]. This lack of hormone therapy efficacy differs from breast cancer, where anti-estrogen therapies have decreased recurrence rates and improved overall survival [36, 37]. This difference is clinically intriguing and may be related to loss of ER expression in metastatic endometrial tumors, which arise from ER-positive primary tumors [38], which indicates the likely emergence of other oncogenic signals. Another possible explanation for the lack of efficacy of adjuvant hormone therapy in type I endometrial cancer is the overall favorable prognosis; the already low recurrence rates may make it difficult to see significant differences in outcomes.

In the metastatic and recurrent setting, several endocrine therapies have been tried, including progestins, tamoxifen, aromatase inhibitors, and fulvestrant with varying response rates of 9–56% [39–50]. Patients with low-grade type I recurrent endometrial tumors are the best candidates for endocrine therapy with progestins being most commonly used [51] and providing variable response rates of between 11 and 37% [52]. ER expressing tumors can also be treated with cycles of tamoxifen and progestins. The concept behind this treatment strategy is that tamoxifen leads to increased PR expression, which enables progestins to act on the tumor cells. The combination of tamoxifen and progestins exhibits similar response rates to progestins alone [53]. Fulvestrant and aromatase inhibitors have shown marginal efficacy in recurrent endometrial cancers [39]. Overall, endometrial cancer treatments reinforce the importance of steroid hormone signaling; however, with quite variable response rates, there is room for improvement in treating metastatic and recurrent endometrial cancer.

## Molecular Details of Estrogen Signaling Through Estrogen Receptor α in Endometrial Cancer

In the early 1960s, work by Elwood Jensen found that E2 was specifically taken up by the immature rat uterus, which led to the hypothesis of an estrophilin that was later termed an estrogen receptor [54]. Twenty years later, the first human estrogen receptor, estrogen receptor α (ER), was cloned [55]. A decade after ER was cloned, a second estrogen receptor with a similar gene structure, named estrogen receptor β, was discovered [56]. ER and estrogen receptor β are steroid hormone receptors that contain four key domains (in order from N-terminal to C-terminal): Activation function 1 (AF-1) domain is involved in the recruitment of cofactors [57]; a C4-zinc DNA binding domain [58]; a hinge region that is required for synergy between AF-1 and activation function 2 (AF-2) in cofactor recruitment [59]; and a ligand-binding domain (LBD) that binds to estrogens with high affinity and recruits cofactors [60], the C-terminus of this domain is referred to as the AF-2 domain and is critical for transcription activation [61]. ER and estrogen receptor β share nearly identical DNA binding domains and more divergent ligand-binding domains with 60% homology [62]. There have been several studies on the expression and role of estrogen receptor β in endometrial cancer with conflicting results (reviewed in [63]), but analysis of the TCGA data [8] suggests that ER is expressed at much higher levels (2.9-fold) on average than estrogen receptor β in endometrial tumors.

Around the same time that estrogen receptor β was identified, an unrelated G protein coupled receptor that bound E2 was also discovered [64, 65]. *GPER*, G protein-coupled estrogen receptor also known as *GPR30*, is a G protein-coupled receptor that resides in the endoplasmic reticulum [66] and has high affinity for E2, but low affinity for the other native estrogens (E1 and estriol (E3)) [67]. *GPER* exhibits low expression in endometrial cancer cells that is 3-fold lower on average than normal endometrial samples [8]. The low expression of *GPER* in endometrial tumors is consistent with a growth inhibitory role in endometrial cancer cells [68]. Of the three estrogen receptors encoded in the human genome, it is thought that the major mediator of pro-growth estrogen signaling in endometrial cancer cells is ER, because of its high expression and mitogenic role in other tissues and cancers.

Estrogen can signal through ER in a genomic and a nongenomic manner. Genomic signaling refers to ER carrying out its typical steroid hormone receptor action of estrogens causing ER to bind the genome and regulate transcription. In nongenomic signaling, ER is bound to the cell surface and when it binds estrogens, it activates other signaling pathways (e.g., cAMP, MAPK) [69]. This allows for more rapid responses than the genomic pathway. Nongenomic signaling can occur in the uterus [70], where it causes the rapid activation of IGF-1 receptor [71]. While the nongenomic actions of ER likely play a role in the normal endometrium and endometrial cancer, it has been shown that RNA synthesis is required for increased protein synthesis in response to estrogens in the uterus [72], and this review will focus on the genomic actions of ER.

**Fig. 2:**
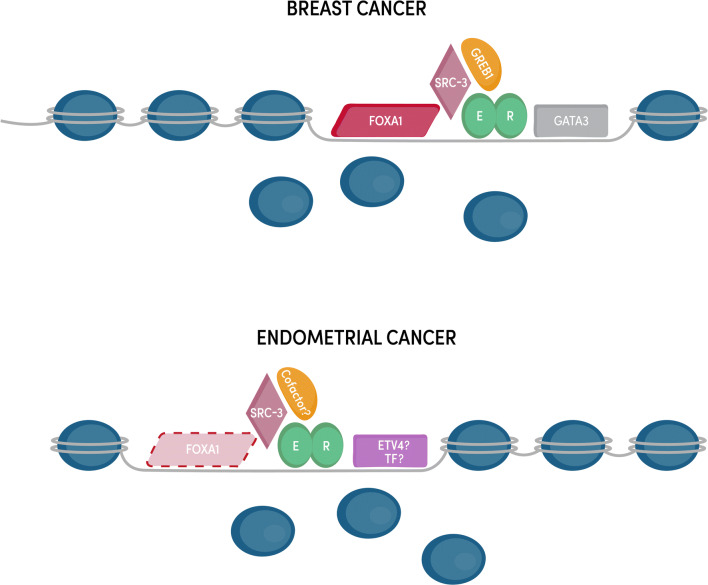
ER’s coregulatory proteins and genomic binding sites differ between endometrial cancer and breast cancer cells. ER binds to mostly different locations in endometrial and breast cancer cells and the majority of these differentially bound loci exhibit differential chromatin accessibility. FOXA1 and GATA3 play a key role in enabling ER genomic binding in breast cancer, but the corresponding transcription factors (TF) in endometrial cancer remain unknown with FOXA1 playing a minor role. While some ER cofactors (e.g., SRC-3) are shared between breast and endometrial cancer, it is unclear if endometrial cancer-specific ER cofactors exist

In the following subsections, we discuss the different molecular aspects of genomic estrogen signaling through ER in endometrial cancer and often compare this knowledge to our understanding of ER’s actions in breast cancer. Much of what we know concerning the molecular details of ER has come from extensive work in breast cancer, where ER has a clear oncogenic role. Despite the similar phenotypic consequences of estrogen signaling, many aspects of ER differ between breast and endometrial cancer and consequently many gaps in our knowledge of ER in endometrial cancer still exist (Fig. 2).

### ER Genome Binding

Estrogen-bound ER is able to form homodimers that bind to estrogen response elements (EREs) in the genome [73]. ER’s preferred sequence for an ERE is 5′-GGTCANNNTGACC-3′, and each monomer of the dimer binds to one half site of the palindromic sequence [74, 75]. Genome-wide analysis of ER binding using ChIP-seq has uncovered thousands of loci bound by ER after E2 induction [76]. The most common motif identified at these loci is the full palindromic ERE; however, the majority of bound sites do not have full palindromic sequence and usually harbor only half EREs instead [77]. The lack of an ERE at the majority of ER-bound sites across the genome was first observed in breast cancer cells but holds true in endometrial cancer cells as well [78]. There are two possible explanations for the non-ERE sites bound by ER. One explanation is that ER can bind to half EREs, which has been observed when an adjacent transcription factor is present (e.g., Sp1, AP-1) [79, 80]. Another explanation is ER binding to loci through protein–protein interactions only, a mechanism referred to as tethering. Tethering has been observed between ER and AP-1 factors as well as CREB1 [81]. Both of these mechanisms likely explain most ER binding sites and while they have not been directly shown in endometrial cancer cells, sequence analysis and modeling of ER-bound loci support these alternative binding modes [78]. The majority of ER-bound loci not containing full EREs is not due to a lack of EREs in the genome. There are approximately 25,000 palindromic EREs in the human genome, but only ~ 10% are bound in a given cell type [77, 78]. The lack of ER binding at most EREs is likely due to the local chromatin environment as the unbound sites are in regions of inaccessible chromatin [77] and harbor DNA methylation [78].

While the properties that govern ER binding site selection are most likely universal across cell types and organisms, ER binds to the genome in a highly cell type-specific manner. ER drives increased proliferation in both breast and endometrial cancer cells; however, ER’s genomic binding targets and the resulting gene expression changes are very different between these cell types. The difference in loci bound by ER in breast and endometrial cancer cells was first observed in cell lines [78, 82] and later confirmed in patient tissues [83] where 15–30% of ER binding sites are shared between breast cancer and endometrial cancer cells. The cell type-specific binding of ER is most likely caused by differences in the chromatin landscape between cell types. As a type I nuclear receptor that is generally restricted from binding the genome in the absence of estrogens, ER is unable to maintain accessible chromatin for its own binding and instead needs to identify binding sites each time it is activated by estrogens. This feature of ER biology manifests in the majority of ER’s binding sites being found in regions of the genome that are accessible prior to E2 treatment based on the presence of DNase I hypersensitive sites [78, 84]. Since the chromatin landscape is inherently different between breast and endometrial cancer cells due to different developmental lineages and a different cadre of transcription factors, this leads to different ER binding profiles and therefore different target genes (Fig. 2).

ER binds primarily to promoter-distal regulatory regions; approximately 95% of ER-bound regions are at least 5 kb from any promoter [85, 86]. Owing to the promoter distal binding of ER, most estrogen induced transcription is driven primarily by gene expression enhancers. These enhancers communicate to target gene promoters through long-range looping interactions. ER is directly involved in these looping interactions, and around 10% of all ER-bound sites have some evidence of looping to another region while ER is bound, based on ChIA-PET data [87]. Consistent with this observation, there are approximately 10-fold more ER-bound sites than genes that change expression in response to E2 treatment in both endometrial cancer and breast cancer cells [78]. The overabundance of ER binding sites could be attributed to not every ER-bound site having the potential to contribute to the acute transcriptional response to estrogens. For example, sites without EREs may not contribute to gene expression, which was postulated based on work with ER DNA binding mutants in the mouse uterus [88]. Correlative studies have identified enhancer RNA production [89] and increases in DNase I hypersensitivity [84] as being associated with expression changes of nearby genes in breast cancer cells, again suggesting that some ER-bound loci have higher regulatory potential than others. Another reason underlying the overabundance of ER binding sites relative to genes is that most genes that are upregulated by E2 harbor multiple ER-bound sites within 100 kilobase pairs of their transcription start sites in both breast and endometrial cancer cells [90]. This observation indicates that many E2 responsive genes could be regulated by multiple ER-bound enhancers. To investigate how multiple ER-bound sites combine to regulate gene expression, Carleton et al. developed enhancer interference, a technique for testing the necessity of enhancers [91]. At each of the three genes tested in endometrial cancer cells, ER-bound sites combine in a synergistic/cooperative fashion to regulate gene expression. At these genes, the presence of a full ERE and being closer to the transcription start site were associated with the importance of a site [90]. While ERE containing enhancers were the most important sites, non-ERE ER-bound enhancers did play a role in supporting the ERE containing enhancers. More work in this area is needed in order to understand the relationship between ER genome binding and gene expression.

### Control of Tissue-Specific Transcriptional Responses to Estrogens

ER’s preferential binding to already accessible chromatin implies the need for other transcription factors to initially bind and create these accessible regions. Pioneer factors are sequence-specific transcription factors capable of binding to condensed chromatin and increasing accessibility at their binding sites, which allows other transcription factors and regulatory cofactors to bind. In breast cancer cells, FOXA1 [92] and GATA3 [93] act as pioneer factors for ER and are responsible for maintaining chromatin accessibility at many of ER’s genomic binding sites (Fig. 2). In endometrial cancer cells, it is unclear which pioneer factors are responsible for enabling ER genomic binding. FOXA1 is expressed in some endometrial tumors and a role for FOXA1 as a pioneer factor in endometrial cancer cells has been proposed [83]; however, FOXA1 overlaps with less than 10% of ER-bound sites in endometrial cancer cells [78] and tumors [83], indicating that the pioneering role is minimal and does not explain most endometrial cancer-specific ER genomic binding. The tissue specificity of ER genomic binding and chromatin accessibility patterns implies an undiscovered endometrial cancer-specific pioneer factor. Motif analysis of endometrial cancer-specific ER-bound sites, along with gene expression analysis, found that the ETS family member, ETV4, may play a role in ER genomic binding and overlaps with ~ 45% of ER binding sites [78]; however, this association has not been functionally evaluated. The mechanisms underlying ER’s unique genomic binding pattern in endometrial cancer cells remain unclear (Fig. 2).

The regulation of ER’s target genes is not only determined by where ER binds but also by the interacting proteins recruited to those sites. ER is capable of binding to cofactors through both the AF-1 and AF-2 domains, acting synergistically in the recruitment of coregulators [94]. Investigation of coregulators of steroid hormone receptors began in the 1990s and has led to the discoveries of coactivators such as the p160 family (known as SRCs or NCOAs) [95–97] and corepressors such as N-CoR and SMRT [98, 99] (Fig. 2). Most of these coregulators are only able to bind to sites when ER is present [100]. Helix 12 of the ligand binding domain is needed for ER’s interactions with many of these cofactors. Crystal structures show that helix 12 lays across the ligand binding pocket when an agonist is bound, generating a surface amenable for the binding of LXXLL motifs found on coregulators [57, 101]. In this manner, estrogen binding and cofactor recruitment are coordinated. Though these coregulators should be able to interact with ER independent of the cell type, cofactors can contribute to the tissue-specific gene regulation by ER through differential expression between tissues.

Differences in cofactor usage could also lead to tissue-specific responses to hormone therapies. Tamoxifen is a commonly prescribed hormone therapy for the treatment of breast cancer and has been effective at reducing recurrence in women with ER-positive breast cancer. Unfortunately, tamoxifen has numerous reported side effects, including a ~ 7-fold increased risk of endometrial cancer in postmenopausal women [102]. In breast cancer cells, tamoxifen acts mostly as an antagonist by binding to the same pocket as E2 and dislocating helix 12 from the ligand-binding domain [103]. However, in endometrial cancer cells, tamoxifen has estrogenic properties and acts as a partial agonist [104]. While the tissue-specific actions of tamoxifen are not fully understood, one model is that the complement of cofactors differs between breast cancer cells and normal endometrial cells and that the binding of some cofactors is blocked by tamoxifen while other cofactors are unaffected [105]. When endometrial cancer arises during tamoxifen treatment, transcription regulation through ER can be altered. ER genomic binding in endometrial cancers that arose after tamoxifen treatment of breast cancer look more like ER binding in breast tumors when compared to ER binding in tamoxifen unassociated endometrial tumors, but the effect is subtle with ER binding across all endometrial tumors clustering together and away from the breast tumors regardless of tamoxifen treatment [106]. The opposing effects of tamoxifen highlight a key difference between breast and endometrial cells that might be due to which coregulators are expressed and used by ER.

While many ER cofactors are known, new methods such as rapid immunoprecipitation and mass spectrometry of endogenous proteins (RIME) have been used to discover other proteins found in the vicinity of ER [107]. RIME is similar to ChIP-seq with the exception that instead of extracting and sequencing DNA after ChIP, proteins are extracted and identified by mass spectrometry. This technique was used to discover a novel cofactor in breast cancer cells, GREB1, which is necessary for stabilizing ER’s interaction with other cofactors [107] (Fig. 2). RIME and similar techniques, such as Bio-ID and APEX [108, 109], will be useful in uncovering endometrial cancer-specific ER cofactors or cofactors that are more commonly used in endometrial cancer cells than breast cancer cells.

### Interplay with Other Steroid Hormone Receptors

Steroid hormone receptors can bind to similar sequences and influence the action of one another [110]. Vahrenkamp et al. recently performed analysis of the association between steroid hormone receptor expression at the mRNA level and outcomes of endometrial cancer patients based on TCGA data [111]. While almost all type I endometrial tumors express ER, higher expression is associated with better outcomes. This association is likely due to high ER expression indicating a hormonally driven tumor that is more differentiated. Not surprisingly, high PR expression is also associated with better prognosis. The expression of ER and PR is highly correlated in endometrial tumors and ER directly regulates PR expression [112]. PR is also able to modulate ER’s gene regulatory role in breast cancer cells as cotreatment with estradiol and a progestin rewires ER genomic binding by causing it to bind to PR-bound sites [113, 114]. A similar relationship might be occurring in endometrial cancer cells where PR blocks ER’s ability to drive growth. The only steroid hormone receptor whose expression is associated with worse outcomes in type I endometrial cancer is glucocorticoid receptor (GR), and this association is only seen in tumors that express high levels of ER [111]. The poor prognosis of tumors with high GR expression is surprising because corticosteroids, which bind to and activate GR, cause growth suppression in the normal uterus [115, 116]. GR-mediated growth suppression is no longer achieved in endometrial hyperplasia and endometrial cancer cells. The loss in growth suppression is accompanied by reprogramming of GR genomic binding in endometrial cancer cells cotreated with E2 and dexamethasone, a synthetic corticosteroid, in which GR binding is moved to sites normally bound by ER [111]. In contrast to findings in endometrial cancer, GR expression is associated with better prognosis in ER-positive breast cancer [117] and GR reprograms ER genomic binding instead [118]. These examples show that steroid hormone receptors can have profound phenotypic and molecular effects on each other; however, more work is needed to uncover how ER, PR, and GR influence one another in endometrial cancer.

## Estrogen Receptor α Mutations Cause Estrogen-Independent Estrogen Receptor α Activity

Mutations in the LBD of ER were initially reported over two decades ago in breast cancer cells [119, 120] and tumors [121]. However, they remained a relatively underexplored phenomenon until recently. The advent of newer generation, deep sequencing technologies has led to the discovery of ER mutations in approximately 15% of metastatic ER-positive breast tumors [122]. Although other ER mutations have been identified at lower frequencies, the most predominant LBD mutations occur within helix 12, mainly affecting three amino acids in that region: L536, Y537, and D538. Studies have shown a clear association between these heterozygous, activating mutations and acquired hormone therapy resistance [122–131]. Interestingly, the mutations are observed in less than 1% of primary breast tumors and are not thought to drive breast tumor initiation [122]. ER mutations are more prevalent in primary endometrial cancer, with 5.8% of tumors harboring an ER mutation [132], representing approximately 3500 new ER mutant endometrial cancer diagnoses in the USA each year.

Advances in genome editing technologies have enabled in depth studies into the consequences of ER LBD mutations. Functional studies in breast cancer indicate these mutations confer estrogen-independent activity to ER, increasing proliferation and driving gene expression in the absence of estrogens, both in vitro and in vivo [122, 125–130]. Mutant ER’s modified/active conformation not only leads to constitutive receptor activity, but also a decreased sensitivity to hormone therapies [122, 123, 129, 130, 133]. The discovery of estrogen-independent ER activity in ER mutant breast cancer is consistent with the observation that endometrial cancer patients with ER mutant tumors have lower body mass indexes than patients with ER wild-type tumors [134], indicating that excess estrogens may not play as large a role in ER mutant tumor formation. Evaluation of an endometrial cancer cell model of the D538G mutation found that mutant ER exhibits estrogen-independent genomic binding as well as an expanded set of genomic binding sites [135]. The constitutively active mutant receptor creates a more open and accessible chromatin landscape in endometrial cancer cells, implying a potential small pioneering role for mutant ER in this disease. Mutant ER causes gene expression changes that are a combination of estrogen-independent regulation of genes normally responsive to E2 and novel regulation of genes that are not responsive to E2. These transcriptional changes are distinct from those observed in ER mutant breast cancer cells; however, similar pathways such as growth and migration are impacted [135, 136]. Understanding the molecular and pathological impact of ER mutations in endometrial cancer will further our knowledge of ER mutant disease and could uncover treatment options for patients with ER mutant tumors, which trend towards worse prognosis [134].

## Outstanding Challenges Surrounding the Role of Estrogen Signaling in Endometrial Cancer

There are several open questions regarding estrogen signaling in endometrial cancer. As discussed above, there are clear signs that ER genomic binding is controlled by different and yet unknown transcription factors in endometrial cancer cells as compared to breast cancer cells. In addition, the cofactors that are utilized in endometrial cancer cells appear to differ from breast cancer cells. Studies into endometrial cancer-specific transcription factors and cofactors are sure to uncover interesting biology and shed light on how cell type-specific gene regulation through ER occurs. The discovery of endometrial cancer-specific ER influencing transcription factors and ER binding cofactors may also uncover clinical vulnerabilities that could be exploited in the treatment of endometrial cancer [137].

The treatment options for endometrial cancer are limited and, until recently, have remained relatively unchanged. According to the FDA, only one hormonally active drug is approved for use in endometrial cancer: megestrol acetate, a progestin. In contrast, over 50 drugs, including several hormone therapies, are approved for use in breast cancer. While progestins are effective in fertility sparing treatment of primary type I endometrial tumors, the recurrence rate is still close to 50% [33]. A greater understanding of how ER functions in endometrial cancer cells could identify additional treatments that work to block ER’s ability to drive growth. In the metastatic setting, there is a need for targeted therapies that effectively treat recurrent tumors and further study of estrogen signaling in endometrial cancer could help identify successful drugs for a subset of metastatic endometrial cancer patients. Patients with ER mutant tumors might require different treatment strategies, as it is unclear if these tumors respond to progestin and if they are inherently more aggressive. There is hope for new therapies being adopted for use in endometrial cancer as pembrolizumab was recently approved for the treatment of microsatellite instable or mismatch repair deficient advanced endometrial cancer.

While excessive estrogen signaling plays a role in endometrial cancer formation, common genomic alterations are also observed. Mutations in *PTEN*, *ARID1A*, *PIK3CA*, *PIK3R1*, beta-catenin, *CTCF*, and *KRAS*, are often found in endometrial tumors with endometrioid histology [8, 138], with a mutation in at least one of these genes found in 94% of tumors. Many of these genes appear to be specific to endometrioid endometrial tumors, as opposed to nonendometrioid endometrial tumors, including transcriptional regulators *ARID1A*, beta-catenin, and *CTCF*, indicating that there may be a connection between these mutations and estrogen signaling. Alterations in signaling pathways can alter ER genomic binding and target genes in breast cancer cells [139, 140], and it would be interesting to discover how endometrial cancer genomic alterations impact how ER regulates gene expression. Along the same lines, ER genomic binding could be a read out for the activity of other signaling pathways and therefore could be used to predict prognosis, as ER binding in breast tumors correlates with aggressiveness of the tumors [141]. An understanding of how endometrial cancer mutations influence estrogen signaling could uncover valuable information concerning crosstalk between environmental and genetic factors of the disease while also informing treatment decisions in endometrial cancer.

Unfortunately, progress has been slow in the clinical management of endometrial cancer and this is correlated with a lack of research funding devoted to understanding and combating the disease. Only 0.3% of the National Cancer Institute’s fiscal year 2017 research funding went towards endometrial cancer research; however, uterine cancer represents 3.6% of cancer cases and 1.9% of cancer deaths. There is currently no Department of Defense Congressionally Directed Medical Research Program (a program that supports research on many different cancer types) that funds endometrial cancer research. Endometrial cancer cases and deaths are on the rise [142], and it is becoming critical that more research, and more funding, are devoted to increasing our understanding of the disease and developing new treatment options for patients with endometrial cancer.
